# Changing the Conversation, Why We Need to Reframe Corruption as a Public Health Issue: Comment on "We Need to Talk About Corruption in Health Systems"

**DOI:** 10.15171/ijhpm.2019.124

**Published:** 2019-11-27

**Authors:** David Clarke

**Affiliations:** World Health Organization (WHO), Geneva, Switzerland.

**Keywords:** Health System Corruption, Health System Strengthening, Public Health Approach, Prevention, Universal Health Coverage

## Abstract

There has been slow progress with finding practical solutions to health systems corruption, a topic that has long languished in policy-makers "too difficult tray." Efforts to achieve universal health coverage (UHC) provide a new imperative for addressing the long-standing problem of corruption in health systems making fighting corruption at all levels and in all its forms a priority. In response, health system corruption should be classified as a risk to public health and addressed by adopting a public health approach. Taking a public health approach to health systems corruption could promote a new paradigm for working on health system anti-corruption efforts. A public health approach could increase the space for policy dialogue about corruption, focus work to address corruption on prevention, help generate and disseminate evidence about effective interventions strategies, and because of its focus on multisectoral action would provide new opportunities for promoting cooperation on anti-corruption work across multiple agencies and sectors. Using a public health approach to tackle health system corruption could help address the current inertia around the topic and create a new positive mindset among policy-makers who would come to see corruption as a manageable public health problem rather than an intractable one.

## Context

On September 23, 2019, heads of state and government representatives issued a political declaration that *reaffirmed the right of every human being, without distinction of any kind, to the enjoyment of the highest attainable standard of physical and mental health* . They also agreed to a dedicated focus on efforts *to achieve universal health coverage (UHC), reaffirming that health is a precondition for and an outcome and indicator of the social, economic and environmental dimensions of sustainable development and the implementation of the 2030 Agenda for Sustainable Development* .^1^

Health system corruption threatens these aspirations. It both undermines the right to health^2^ and creates barriers to efforts to achieve UHC. Health systems corruption negatively impacts on the right to health and efforts towards UHC by blocking people’s access to quality health services and safe and effective medicines, while also undermining systems for financial risk protection.^3^

As Hutchinson et al^4^ point out, the health sector is particularly vulnerable to corruption. Health system corruption occurs worldwide and is a long-standing problem in both developed and developing countries. The consequences of healthcare corruption are significant. Each year, it contributes to an estimated 140 000 child deaths, global health expenditure losses estimated at 6.19% of total global health expenditure^5^ and public procurement losses for medicines and vaccines in the range of 10%-25%.

However, despite the negative impact of corruption on the health of individuals and efforts towards the global UHC goal, the issue has long been a taboo topic and languished in policy-makers “too difficult basket.” Hutchinson and colleagues^4^ posit several factors that contribute to this state of affairs. Another major factor is the fact that corruption is a collective action problem.^6-11^ When corruption is systemic and widely perceived to be the norm, it is difficult for policy-makers to see it as a policy problem that is amenable to solutions.

The recent United Nations political declaration on UHC declared that fighting corruption at all levels and in all its forms is a priority.^1^ This high-level political commitment creates an imperative for a new approach to tackling health system corruption. But the question is how to implement this commitment in practice? I argue that a public health paradigm should be used, classifying corruption as a public health problem and applying public health approaches to addressing it.

## Taking a Public Health Approach to Addressing Health System Corruption

A public health model is a conceptual approach that has widespread acceptance across many disciplines, including health, education, and welfare.^12^ Public health models aim to prevent problems from occurring in the first place by targeting key risk factors or determinants and addressing these at a population-level. Rather than merely accepting or reacting to corruption, the starting point for a public health approach to anticorruption would-be the belief that corrupt behaviours and its causes can be prevented.

A public health approach to corruption would involve promoting an understanding of health system corruption as a complex problem with a series of determinants. Public health commonly uses social-ecological models to develop such an understanding by examining the various factors that contribute to behaviours that pose a risk to health. In the case of health system corruption, this model would include examining the influence of social, cultural, political, institutional and environmental factors that contribute to corruption in health systems.

In practice, applying a public health approach to addressing the issue of corruption would involve four steps.

First, better defining the nature of the problem through the systematic collection of information about the magnitude, scope, characteristics and consequences of corruption. Second identifying risk and protective factors that influence corruption. This step would involve examining why corruption occurs in terms of the causes and correlates of corruption, the factors that increase or decrease the risk of corruption and the factors that might be modified through interventions. The third step would be to develop and test corruption prevention strategies and programs. With the information gathered in the previous step, intervention strategies can be designed to target risk and protective factors. Last, a public health approach would also involve integrating evaluation into prevention programs to obtain evidence to compare the effectiveness of different programmatic approaches.

## The Benefits of Using a Public Health Approach

Hutchinson et al^4^ argue that the tropic of health system corruption has failed to engage global; policy-makers who have put the topic in the “too difficult” tray. The recent statement in the United Nations political declaration about the importance of working to address corruption creates a new impetus for solving this problem and for finding new ways for creating space for policy dialogue and policy action to help implement this commitment. The first benefit offered by a public health approach is its potential to create this space by reframing the problem of corruption in public health terms.

Katikireddi et al^13^ advocate for policy reframing as a deliberate strategy to promote health policy dialogue and policy action. They argue that the policy framing directly impacts on the extent to which policy-makers are open and responsive to work on a specific policy issue. Public health practitioners have long used the technique of reframing. One example is how the public health community reframed smoking-related disease as a public policy problem with legal and fiscal solutions targeting tobacco industry behaviour and tactics.^12^

How would such an approach be applied to efforts to address corruption? The current policy framing of health system corruption focuses on prohibition, criminalisation and punitive measures. Institutional and legal reforms and capacity building are standard policy responses. Key actors in these policy responses are anti-corruption and law enforcement agencies rather than health agencies.

In contrast, a public health approach would frame efforts to address corruption by focusing on how anti-corruption efforts contribute to maximising the utility of health resources to improve health services and health status as part of broader health system strengthening efforts aimed at realising the right to health and achieving UHC.

Public health framing for addressing health system corruption would acknowledge that all societies and country contexts are vulnerable to corruption; acknowledging this fact, while proactively building institutional capacity and ensuring measures to inhibit/prevent the development of corruption as part of wider efforts to strengthen health systems significantly increase the policy space for health agencies to work on corruption. While punitive/remedial actions are often still required, an increased focus on public health and prevention shifts the dominant focus of anti-corruption efforts from reactive measures towards creating innovations in prevention, including risk management, and opening new avenues and policy space for addressing what a politically sensitive issue for many governments.

Accordingly, the second benefit of taking a public health approach to corruption is the core emphasis in a public health approach on prevention. A public health framework for preventing corruption would involve work on prevention of corruption on three levels: Primary prevention focused on developing strategies aimed at avoiding and mitigating risk. Secondary prevention (early intervention), this refers to programs that require early detection of corruption risk or early manifestations of corruption. Tertiary prevention (response or intervention) these would be responses set in motion after a corrupt act has occurred. They would aim to reduce the consequences and impacts of an event and help prevent and deter recurrence.

The third advantage of a public health approach is the public health emphasis on integrating evaluation into prevention programs to obtain vital evidence to compare the effectiveness of different anti-corruption programs and strategies. In the health system corruption area, there is a significant body of literature describing the problem of health system corruption. However, there is a considerable know-how gap, with far less literature and guidance about what to do to address corruption and how to implement and prioritise anti-corruption activities.^4^ Employing a public health approach has the potential to close this gap and create a new body of evidence for guiding future anti-corruption efforts.

The fourth advantage of a public health approach is its potential to build anti-corruption coalitions across sectors. An example is the recent formation of the Global Network on Anti-Corruption, Transparency and Accountability led by the World Health Organization, the Global Fund, and the United Nations Development Programme in February 2019.^14^ The foundation for the work of the coalition is an emphasis on a public health approach focusing on risk management, prevention and health system strengthening efforts to support efforts towards UHC.

## Conclusion

Existing anti-corruption approaches cannot effectively solve the healthcare corruption problem. Instead, corruption needs to be approached as a complex phenomenon with multiple causes. This is where a public health approach comes in. A public health approach to corruption seeks to support efforts towards improving health services and population health status by identifying and addressing the underlying risk factors that increase the likelihood of health system corruption and create barriers to delivering effective health services and products. This is quite a different approach to traditional approaches to curbing corruption that has focused on prohibition, criminalisation and punitive approaches.

By taking a public health preventive approach to addressing the entry points where corruption could occur, much can be done to produce a paradigm shift in how health systems, as well as development partners, address corruption. All societies and country contexts are vulnerable to corruption; acknowledging this, proactively building institutional capacity and ensuring measures to inhibit/prevent the development of corruption is an integral part of broader reforms aimed at delivering better health. It enables the maximisation of health benefits from public resources and builds public trust in the system. While punitive/remedial actions are often still required, an increased focus on prevention shifts the dominant focus from reactive measures towards creating innovations in prevention, including risk management, and opening new avenues for addressing a taboo politically sensitive issue.

A key strength of a public health approach is that it draws on input from diverse sectors including health, anti-corruption, justice, policy and civil society. Promoting collective action on the part of these stakeholders can help in addressing problems like corruption.

Taking a public health approach to health systems corruption could promote a new paradigm for working on health system anti-corruption efforts. I argue that using a public health approach to tackle health system corruption emphasising corruption as a problem that can be understood and prevented, could help address the current inertia around the topic and create a new positive mindset among policy-makers who would come to see corruption as a manageable public health problem rather than an intractable taboo issue.

## Ethical issues

Not applicable.

## Competing interests

Author declares that he has no competing interests.

## Author’s contribution

DC is the single author of the paper.
